# Kinesiotherapy With Exergaming as a Potential Modulator of Epigenetic Marks and Clinical Functional Variables of Older Women: Protocol for a Mixed Methods Study

**DOI:** 10.2196/32729

**Published:** 2021-10-13

**Authors:** Patrícia Paula Bazzanello Henrique, Fabrízzio Martin Pelle Perez, Osvaldo Henrique Cemin Becker, Ericles Andrei Bellei, Daiana Biduski, Arthiese Korb, Daniela Pochmann, Caroline Dani, Viviane Rostirola Elsner, Ana Carolina Bertoletti De Marchi

**Affiliations:** 1 Faculty of Physical Education and Physiotherapy University of Passo Fundo Passo Fundo Brazil; 2 Department of Physiotherapy Regional Integrated University of High Uruguay and Missions Erechim Brazil; 3 Institute of Exact Sciences and Geosciences University of Passo Fundo Passo Fundo Brazil; 4 Graduate Program in Biosciences and Rehabilitation Porto Alegre Institute of the Methodist Church Porto Alegre Brazil; 5 Graduate Program in Biological Sciences: Physiology Federal University of Rio Grande do Sul Porto Alegre Brazil

**Keywords:** elderly women, exergame, epigenome, cognition, kinesiotherapy

## Abstract

**Background:**

Kinesiotherapy is an option to mitigate worsening neuropsychomotor function due to human aging. Moreover, exergames are beneficial for the practice of physical therapy by older patients. Physical exercise interventions are known to alter the epigenome, but little is known about their association with exergames.

**Objective:**

We aim to evaluate the effects of kinesiotherapy with exergaming on older women’s epigenetic marks and cognitive ability, as well as on their clinical functional variables. Our hypothesis states that this kind of therapy can elicit equal or even better outcomes than conventional therapy.

**Methods:**

We will develop a virtual clinic exergame with 8 types of kinesiotherapy exercises. Afterward, we will conduct a 1:1 randomized clinical trial to compare the practice of kinesiotherapy with exergames (intervention group) against conventional kinesiotherapy (control group). A total of 24 older women will be enrolled for 1-hour sessions performed twice a week, for 6 weeks, totaling 12 sessions. We will assess outcomes using epigenetic blood tests, the Montreal Cognitive Assessment test, the Timed Up and Go test, muscle strength grading in a hydraulic dynamometer, and the Game Experience Questionnaire at various stages.

**Results:**

The project was funded in October 2019. Game development took place in 2020. Patient recruitment and a clinical trial are planned for 2021.

**Conclusions:**

Research on this topic is likely to significantly expand the understanding of kinesiotherapy and the impact of exergames. To the best of our knowledge, this may be one of the first studies exploring epigenetic outcomes of exergaming interventions.

**Trial Registration:**

Brazilian Clinical Trials Registry/Registro Brasileiro de Ensaios Clínicos (ReBEC) RBR-9tdrmw; https://ensaiosclinicos.gov.br/rg/RBR-9tdrmw.

**International Registered Report Identifier (IRRID):**

DERR1-10.2196/32729

## Introduction

### Background and Rationale

Human aging is a natural process that causes a series of neuropsychomotor changes, such as decreased muscle strength, proprioception, balance, and cognition [1,2]. An option to mitigate this deterioration is kinesiotherapy, a therapeutic exercise that trains body movements and postures in a planned and systemic way, improving the patient’s functional capacity, autonomy, and well-being [3].

Neuroprotective effects of physical exercise and the improvement of its clinical and functional outcomes are partly associated with the modulation of epigenetic marks, in both preclinical and clinical studies [4-9]. Epigenetics is the study of heritable changes in gene function that do not entail a change in DNA sequence [10]. Epigenetic marks are modulated by gender and time of the day [11]. Especially for older women, physical exercise has a role in improving health conditions [12-14], which is even indicated in biomarkers [15].

Older people face barriers to take part in exercise, but they identify positive aspects of strength and balance activities [16,17] and report positive perceptions about implementing technology to exercise [18]. Hence, appropriate programs and interventions based on education and training can help overcome the barriers [16]. Likewise, motivation and confidence for older persons can enable better performance in physical exercise [19]. In all these aspects, appropriate interactive technologies can have a facilitating role [20].

Virtual games are beneficial in physical therapy practice, particularly in the rehabilitation of functional balance, postural skills, and motor skills [21-23]. Exergames are virtual games that can capture the user’s real movements and promote physical activity, especially for older people [24]. This kind of game encourages the use of body movements to interact with the virtual scenario in a stimulating and integrative way, generating an enriched setting and greater motivation for progressing motor skills [25,26]. Game-based therapy can also be an alternative approach to improve cognitive functions, with improvements in different cognitive domains, such as executive function, visuospatial attention, verbal memory, and working memory [27]. Furthermore, rehabilitation exercises with exergaming have become an option for rehabilitation programs and have been adopted by physical therapists [28].

### Aims and Hypothesis

There is emerging evidence that physical exercise interventions can alter the epigenome, and the outcomes could be related to specific pathways [29,30]. However, to the best of our knowledge, there is still no evidence in the literature addressing the association of exergaming with epigenetic marks. Given the heterogeneity and complexity of the existing literature, more research is required in this emerging area to identify epigenetic marks that could serve as indicators of exercise adaptations [29]. In this perspective, this study aims to evaluate the effects of kinesiotherapy with exergaming on older women’s epigenetic marks and cognitive ability, alongside the evolvement of clinical functional variables. We hypothesize that this kind of therapy can elicit satisfactory results, with similar or even better outcomes than those of conventional therapies.

## Methods

**Figure 1:**
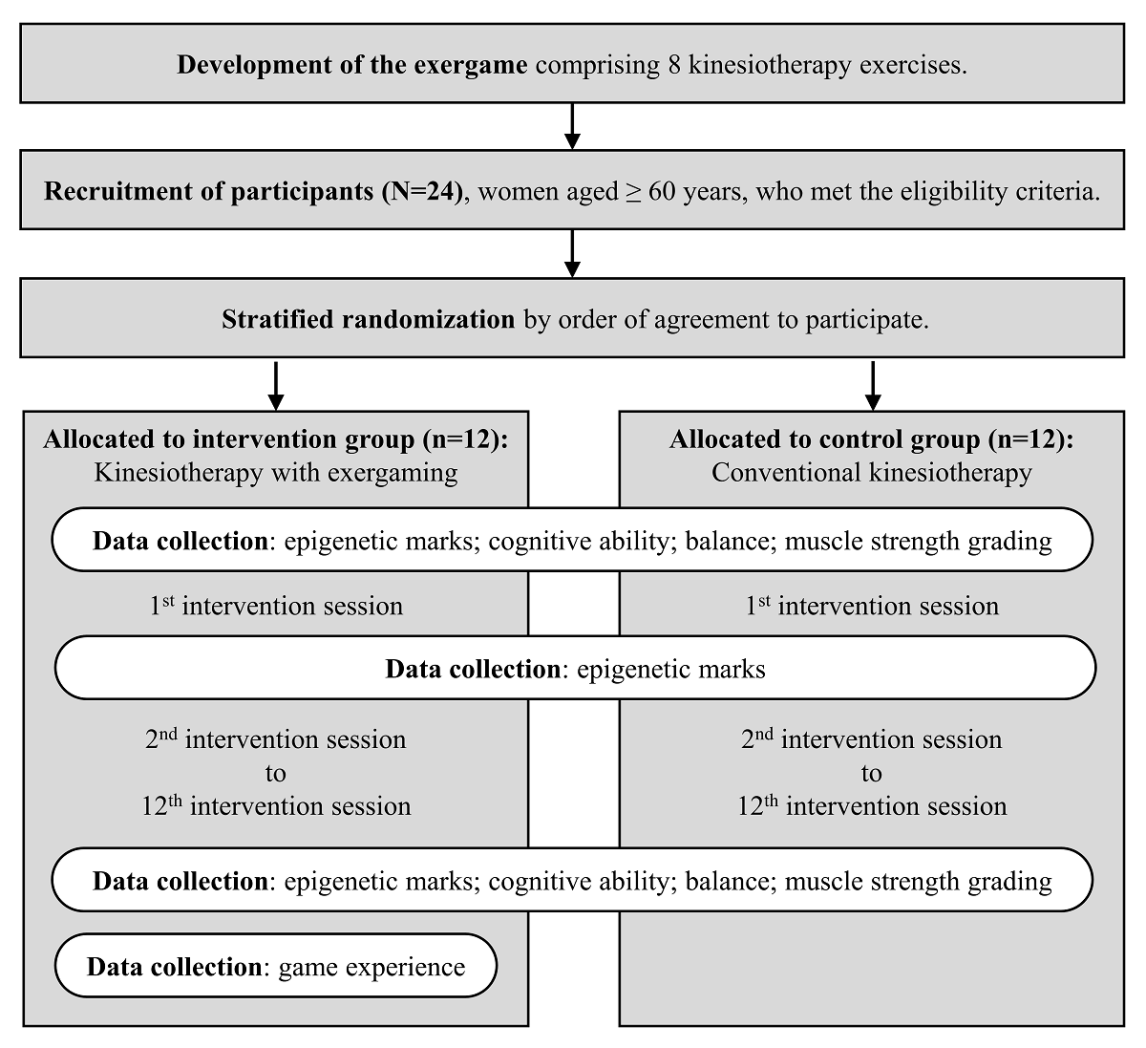
Study procedure flowchart.

This is an applied study with software development and analysis of its effects through a controlled, parallel, two-armed, open-label, randomized clinical trial. Figure 1 shows the procedure flowchart.

### Exergame Development

Exergames that require an individual to repeatedly reach for an object do not use the full potential of exergaming as a rehabilitation tool [31]. Taking this into account, we will develop an exergame with a virtual scenario based on a physiotherapy clinic. In this environment, 8 types of exercises practiced in kinesiotherapy sessions will be available, including squats, horizontal shoulder abduction, hip abduction, diagonal movement of the upper limb (Kabat diagonals), plantar flexion, elbow extension, elbow flexion, and horizontal shoulder adduction. Reference images from real environments will be used to model the setup and all interactable objects used by the avatars.

The clinic scenario will have windows that show an outdoor view with trees and clouds reacting to the wind. The lighting and animations will also change according to the day and time the user is playing, whether day or night. A male and a female character (avatar) will be available to play, selected according to the gender registered for each user. The objects that the avatar will interact with when performing an exercise will be automatically selected according to the type of exercise. Four camera positions will be implemented in the game. The game will have a menu with options for registration, settings, and types of exercise. During the execution of the exercises, information on the number of repetitions and completed series will appear on the screen, in addition to the execution time.

The exergame will be developed using the programming language C# (Microsoft Corporation), with the game engine Unity 3D (Unity Technologies). Both the scenario and the physiotherapy equipment will be modeled on Blender (Blender Foundation), open-source 3D creation software for animation, simulation, rendering, composition, and motion tracking. The characters will be created in Adobe Fuse (Mixamo), which also offers prebuilt animations and an autorigging tool in the mesh of the created characters. Some other tools, such as Audacity, for editing the sound effects, and Paint.NET (dotPDN LLC) for editing the interface sprites and object textures, may be used in the development of the game.

The participant plays the exergame by watching their avatar on a 42-inch monitor. The motion capture will use an Astra Pro depth sensor (Orbbec 3D Tech Intl Inc) and the software Nuitrack (3DiVi Inc), which analyzes the data obtained by the sensor to identify the user’s movements. To identify the completed repetitions and errors, the game will have rigidbody colliders in the avatar and in the scenario, passing the avatar positions to the scripts that evaluate whether the movement was correct or not.

One of the advantages of the proposed exergame over conventional therapy is that the game is more stimulating and has a joyful purpose, which provides a form of biomechanical biofeedback—feedback on measurements of movement, balance, postural control, and force output [32]. The exergame will have a dynamic virtual environment to engage participants, which will include elements such as background music, object sounds, avatars demonstrating movements as a form of instruction, sound and visual effects to reward achievements and exercise conclusions, scores, individual progress history, automatic counting of series and repetitions, the possibility of competition between participants, and control of times and turns. Hence, we expect to enhance the gains achieved during therapy, throughout the sessions, and perhaps even in a shorter period, as achieved by Henrique et al [21].

### Sample, Eligibility, and Allocation

We will recruit 24 participants, exclusively women aged 60 years and over, at the School Clinic of Studies and Professional Practices of the Regional Integrated University of Upper Uruguay and Missions, which is located in Erechim, Rio Grande do Sul, Brazil. The sample size was based on the studies that used a similar methodology to assess the effect of exercise on epigenetic marks and the effect of improved functional mobility induced by running in the older women, related to epigenetic marks [8,9], considering the global H4 histone acetylation levels variable from earlier studies [33,34]. Sample size was calculated using G*Power software [35] considering an effect size of 1.3, two tails, an error probability of .05, and a power of 0.8.

After the invitation to take part in the study, the participants will sign a consent form and complete a demographic questionnaire. The following inclusion criteria will be verified: age 60 years or older; ability to ambulate effectively; absence of any reported diagnosis of neurological, cardiac, or any other disease that restricts physical exercise; absence of depression confirmed by the Geriatric Depression Scale—Short Form [36]; cognitive ability confirmed by the Mini-Mental State Examination, Brazilian version [37]; and acceptance of the commitment not to undergo any other physical therapy during the study.

The sample will have stratified randomization for 2 groups with 12 participants each. Patients will be selected by order of agreement to participate in the study until the number of patients stipulated for either of the groups is reached. The intervention group will practice kinesiotherapy exercises with the exergame, whereas the control group will practice conventional kinesiotherapy exercises without exergaming. Owing to the nature of the intervention, masking of the physical therapists and participants is not possible. However, data will be collected and analyzed by professionals masked to the allocation of the groups.

### Outcome Measurements

Throughout the clinical trial, we will assess different data for two assays. The first assay will assess the acute and late effects on the participants’ epigenetic marks (primary outcome) as well as the cognitive ability (secondary outcome). Meanwhile, the second assay will assess the participants’ balance and muscle strength grading (primary outcomes), in addition to the postgame experience (secondary outcome).

#### First Assay

Epigenetic marks will be assessed with blood tests. The samples will be collected by qualified and verified professionals, from veins located in the antecubital area, in a sanitized and cool place previously prepared for the procedure. Furthermore, 20 mL blood will be drawn using disposable syringes and needles. The venipuncture procedure will be applied and all national biosafety procedures will be followed. Initially, the blood will be divided into two tubes: a tube containing a separating gel that will receive 5 mL blood to obtain the serum. The other tube will contain the EDTA anticoagulant and will receive 15 mL blood. Then, Histopaque will be added in a 1:1 ratio to the tube with EDTA, which will then undergo a series of centrifuges for the extraction of plasma and peripheral blood mononuclear cells. The material will be stored in appropriate containers at −20 °C. Biomarker analysis will be performed using commercially available assay kits, according to the manufacturer’s guidelines.

The types of indicators that will be analyzed using biomarkers include plasma levels of the brain-derived neurotrophic factor (BDNF), a member of the neurotrophin family that has an important role in neuroplasticity and is associated with the process of memory and learning. We will also analyze the global acetylation levels of histones H3 and H4, which are epigenetic markers associated with increased transcriptional activity and gene expression. A previous study [9] identified that a single exercise session induced a state of global DNA hypomethylation but did not alter the global acetylation levels of H4 histone. However, these markers were not modified after more sessions, suggesting that epigenetic modulation in response to physical exercise is transient, which justifies an analysis comparing the acute and late effects.

Cognitive ability will be assessed using the Brazilian version of the Montreal Cognitive Assessment protocol [38]. This is a brief screening tool that assesses a wide range of cognitive functions, such as executive functions, visuospatial skills, memory recovery, digits, sentences, abstract reasoning, and orientation. The test time is estimated at 20 minutes and the maximum possible score is 30 points. The cutoff score is 26 points.

#### Second Assay

The balance will be assessed using the Timed Up and Go test, which determines fall risk and measures, in seconds, the time taken by an individual to stand up from a chair, walk a distance of 3 m, turn, walk back to the chair, and sit down. For healthy older adults, the ideal time for the test is 10 seconds. When performed between 11 and 20 seconds, it indicates that the individual may have some disability or fragility, which is considered partial independence and low risk of falls. When the test is performed with a time ≥20 seconds, the individual is classified as dependent and has a considerable deficit in physical mobility and a high fall risk [39,40].

Muscle strength grading will be assessed using a push-pull hydraulic dynamometer. This is a device that operates on the principle of traction and compression. An external force is applied to the dynamometer and its spring is tensioned, moving an indicator of the amount of static force applied.

Game experience will be assessed using the postgame module of the Game Experience Questionnaire [41]. It measures players’ experience after the gaming session and any aftereffects (eg, returning to reality, fatigue, pride, guilt) using 17 statements with a semantic differential scale response to indicate the level of agreement.

### Procedure and Follow-up

During the study, participants in both groups will not be allowed to perform any other type of physical therapy. Intervention sessions for both groups will be carried out twice a week. Each session will last an hour, for 6 weeks, resulting in 12 sessions. This time frame is based on similar studies of Duque et al [42] and Park and Yim [43]. The set of kinesiotherapy exercises was designed based on exercises from successful studies on older rehabilitation [21,44,45], whose movements can be captured by the motion sensor used in the exergame.

We will request all participants to attend sessions wearing dark workout clothes to standardize them and also facilitate body detection by the motion sensor. Each participant will be placed in a room with the setup ready. For the control group, it will be a conventional physiotherapy clinic with the necessary equipment for the exercises. For the intervention group, the environment will contain the same equipment, along with the exergame setup consisting of a 42-inch monitor and the motion sensor.

Both groups will be accompanied by 2 physical therapists throughout the sessions. The professionals will provide instructions and adjust the equipment used in the sessions following a standardized procedure protocol to avoid any type of bias. In the event of an incident (eg, harmful movement, injury, or misuse of equipment), the professional will intervene, pausing the session until the problem is fixed. The session will resume as soon as possible so that the participant can complete the remaining time.

The exercises will use dumbbells, TheraBands, and Swiss ball accessories. The weight of the dumbbells and resistance level of the TheraBand will be proportionally tailored to each participant. We will use the Modified Borg Dyspnea Scale [46] to verify the participant’s perception of effort and to define the accessories with an inversely proportional level of difficulty of use. In the first intervention session, for all participants, we will provide a 1-kg dumbbell and a light-resistance TheraBand. Then, at the end of each session, the physical therapists will evaluate each participant’s perception of effort in performing the exercises using her respective accessories. This will support the choice of the dumbbell weight and the TheraBand resistance that the participant will use in the following session. Different Swiss balls will be used, depending on the height of the participant: the small version of 55 cm for people with a height of 160–174 cm, and the average version of 65 cm for people with a height of 175–195 cm.

Performed individually, the kinesiotherapy session will start with a warm-up exercise (10 minutes), using a horizontal exercise bike. After that, the following 8 specific exercises will be performed:

1. Squatting with dumbbells in hands, for 3 minutes.
2. Horizontal shoulder abduction with elbows in extension holding the TheraBand and sitting on the Swiss ball, for 3 minutes.
3. Hip abduction with the TheraBand around the lower limbs, for 6 minutes.
4. Kabat diagonals (adapted) with the patient sitting on the Swiss ball, for 6 minutes.
5. Plantar flexion for calf with dumbbells in hands, for 3 minutes.
6. Horizontal shoulder adduction with elbows in extension holding the TheraBand and sitting on the Swiss ball, for 3 minutes.
7. Elbow flexion for biceps using TheraBand and sitting on the Swiss ball, for 3 minutes.
8. Flexion of hamstring, quadriceps, iliopsoas, and gastrocnemius, performed during a series of 30 seconds for each muscle group, repeatedly, for 6 minutes.

After the strength exercises, the participants will perform cervical spine stretching, lateral and posterior trunk stretching, hamstring stretching, and quadriceps, iliopsoas, and gastrocnemius exercises. The exercises will be performed in a series of 30 seconds, resulting in 10 minutes of activities. Among the different types of exercises, there will be intervals of at least 30 seconds for rest and adjustment of game settings.

The intervention group will perform the same procedure and use the same kinesiotherapy equipment as the control group but playing the exergame with its 8 specific exercises as the basis of the session. The exercises will always be performed in the same order and duration, from the first to the last session. During the intervention, there will be a tolerance of only 2 absences per participant, which must be recovered immediately in the same week of the absence. Otherwise, the participant will be removed from the study.

### Data Collection

Blood test values to assess epigenetic marks will be collected from all participants at three stages: at baseline; after the first session of the intervention, to verify the acute effects; and after the complete intervention, to verify the late effects. Cognitive ability, balance, and muscle strength values will also be collected from all participants at two stages: at baseline and after the complete intervention. Game experience data will be collected exclusively from the participants who played the game (intervention group) after the intervention is completed.

### Statistical Analysis

Basic quantitative data will be determined by mean, SD, and median. Categorical data will be determined by simple frequency. Numeric data will be analyzed using statistical software SPSS, version 22 (IBM Corporation). The normality of the data will be tested using the Shapiro-Wilk test. Moreover, depending on the distribution, we will use one-way factor analysis of variance with Tukey post hoc (parametric data), or Kruskal-Wallis test (nonparametric data). To evaluate the main outcomes of the study, a generalized estimation equation with gamma distribution will be performed. To correlate the data, we will apply Spearman and Pearson analyses considering a 95% CI (*P*<.05). All analysis techniques can be adjusted or modified, if required, by the characteristics of the collected data.

## Results

The project was funded in October 2019. Game development took place in 2020. Patient recruitment and clinical trial are planned for 2021.

## Discussion

The therapeutic resources used in physiotherapy, including the use of virtual reality technologies applied to kinesiotherapy, have been highlighted in recent years. Moreover, physical therapy has used technology to make interventions more engaging, convenient, and fun. Among the many possibilities offered by technology, exergames can be a powerful tool, with opportunities for users to participate in different rewarding experiences, as they rely on motivational aspects [25] and have adaptive strategies to each user, regardless of their motor and cognitive skills [47]. Exergames are a safe, feasible, and beneficial tool for physical exercises by older people [48]. They can combine physical activity, game dynamics, challenges, and achievements in a comfortable environment, merging real-world elements and virtual contents in a single view [48,49]. O’Loughlin et al [50] stated that exergaming is a healthier alternative to sedentary behavior, due to higher energy expenditure and improved physical fitness. In addition, exergames may improve balance, motor coordination, and muscle strength, increasing adherence to exercise in the older population while keeping them physically and socially motivated [25,51-53].

Technological solutions are flexible and have the potential to be accessible, reaching more users at the same time [54]. They also introduce a new style of rehabilitation and practice of physical activities, allowing health professionals to remotely assess the progress of patients and adjust the training strategy accordingly, offering customized experiences related to each user profile, while motivating patients by engaging family members, caregivers, and friends [55,56]. However, a considerable part of the exergames used in physiotherapy is derived from commercial sources [52,57-60], generally not designed exclusively for the older population, or are not based on specific therapy protocols [61]. Moreover, new studies should include games that can target multiple physical functions to determine the extent to which exergaming can contribute to keeping older adults active and healthy [31]. Therefore, it is plausible that an exergame developed from a specific kinesiotherapy model designed exclusively for the older population can elicit better outcomes, which justifies the exergame that we will develop.

We expect that both groups have improvements in muscle strength, balance, and cognitive ability, as both of them will exercise. However, due to the motivational and enriching aspects of the exergame, we expect the intervention group to show a significant improvement compared to the control group. We assume that improvement in functional clinical outcomes in the exergame group will be associated with increased BDNF levels and global acetylation of histones H3 and H4. Strategies that modulate BDNF and acetylation levels might be indicated to improve memory in the older population. Many studies have already shown that exercise is a strong epigenetic modulator that increases BDNF levels [7-9,33,62].

Research on this topic is likely to significantly expand the understanding of kinesiotherapy and the impact of exergames for older women. To the best of our knowledge, this may be one of the first studies exploring epigenetic outcomes of exergaming interventions.

## Appendix

Multimedia Appendix 1 Peer review report from the grant agency.

## Abbreviations

BDNF: brain-derived neurotrophic factor
FAPERGS: Research Foundation of the State of Rio Grande do Sul
