# Supplementary material for: Kinesiotherapy With Exergaming as a Potential Modulator of Epigenetic Marks and Clinical Functional Variables of Older Women: Protocol for a Mixed Methods Study
Source: JMIR Res Protoc. 2021 Oct 13;10(10):e32729. doi: 10.2196/32729 (PMC8552101; doi:10.2196/32729)
Supplement: Multimedia Appendix 1 [file resprot_v10i10e32729_app1.pdf]

FAPERGS
Fundação de Amparo à Pesquisa do Estado do Rio Grande do Sul

Formulário de Pareceres FAPERGS
Parecer entregue em: 23/04/20, 13:41
Documento gerado em: 23/08/2019, 12:36

Consultor: Consultor
Instituição do Consultor: Instituição
Projeto: Efeitos de um protocolo de intervenção cinesioterapêutica com o uso de exergame como potencial agente modulador de marcadores epigenéticos e desfechos clínico-funcionais em idosas
Protocolo: 42338.540.18995.12072019
Edital: EDITAL FAPERGS 05/2019 - PROGRAMA PESQUISADOR GAÚCHO-PQG

1: Excelência da Proposta:

Excelência da proposta quanto aos seguintes aspectos: qualidade e originalidade do projeto; avanço esperado em relação ao estado da arte; efetividade da metodologia proposta.
Valor entre 0 e 10: [9]

2: Experiência do Coordenador:

Experiência prévia do coordenador na área do projeto de pesquisa, considerando sua produção científica e tecnológica relevante, bem como sua contribuição na formação de recursos humanos desde 01/01/2014 (*)
Valor entre 0 e 10: [7,92]

3: Adequação do orçamento:

Adequação do orçamento aos objetivos, atividade e metas propostas.
Valor entre 0 e 10: [9]

4: Potencial de Impacto:

Potencial de impacto dos resultados do ponto de vista técnico-científico, de inovação, difusão, sócio-econômico e ambiental.
Valor entre 0 e 10: [9]

5: Detalhar os aspectos positivos e negativos da proposta (este parecer poderá ser enviado ao proponente, porém com sigilo do nome do avaliador) e justificar eventuais cortes no orçamento proposto e/ou despesas vedadas pelo edital:

O projeto tem alto grau de contribuição social. A metodologia é muito adequada e bem descrita, inclusive com protocolos experimentais e mecanismos de suporte. Foi encaminhada para comitê de ética específico. A coordenadora tem grande experiência com coordenação de projetos nacionais e também com a participação em outros projetos. O estado da arte é relativamente atual, com alguns poucos trabalhos de data mais antiga. A proponente solicita cerca de 20% do valor total do projeto para taxas de publicação em periódicos e tradução, indicados como consumo, mas os mesmos são serviços de terceiros.

6: Baseado na sua avaliação, seu parecer é:

a)RECOMENDADO
b)NÃO RECOMENDADO
c)RECOMENDADO PRIORITARIAMENTE
d)RECOMENDADO COM RESTRIÇÕES

Categoria	Total parcial
1 - Excelência da Proposta:	36,00
2 - Experiência do Coordenador:	31,68
3 - Adequação do orçamento:	9,00
4 - Potencial de Impacto:	9,00
Total Final	85,68
Média Final	8,57

Authors' transcript of the review report to the grant proposal that was approved

(*logo*)<br>FAPERGS<br>Research Foundation of the<br>State of Rio Grande do Sul

Report from the granting agency FAPERGS
Document printed on April 23, 2020, 01:41 pm
Reviewers' report submitted on August 23, 2019, 12:36 pm

Grant reviewers (blind for the authors)
Grant reviewers' affiliation (blind for the authors)
Project name: Effects of a Protocol using Kinesiotherapy with Exergaming as a Potential Modulator of Epigenetic Marks and Clinical Functional Variables on Elderly Women
Protocol ID: 42338.540.18995.12072019
Call for grant proposals: Call FAPERGS 05/2019 – Gaúcho Researcher Program

1: Excellence of the proposal:
Excellence of the proposal regarding the following aspects: quality and originality of the project; expected advances to the state of the art; effectiveness of the proposed methods.
Rating value, from a 0 to 10 scale: [9]

2: Experience of the coordinator:
Previous experience of the coordinator in the area of the research project, considering his/her relevant scientific and technological production, as well as the contribution to the education of human resources since 01/01/2014 (*)
Rating value, from a 0 to 10 scale: [7.92]

3: Adequacy to the budget:
Adequacy of the budget to the objectives, activities and goals proposed in the research project.
Rating value, from a 0 to 10 scale: [9]

4: Potential for impact:
Potential for impact of results from the technical-scientific, innovation, diffusion, socio-economic and environmental point of view.
Rating value, from a 0 to 10 scale: [9]

5: Detail the positive and negative aspects of the project proposal (this opinion may be sent to the proponent, however the name of the appraiser is confidential) and justify any cuts in the proposed budget and / or expenses prohibited by the call for grant proposal:
The project has a high degree of social contribution. The methodology is very adequate and well described, including experimental protocols and support mechanisms. The proposal was also submitted to the specific ethics committee. The coordinator has great experience with coordinating national projects and also participating in other projects. The state of the art is relatively new, with a few studies with older publication dates. The proponent requests about 20% of the total value of the project for publication fees in journals and for translation, indicated as consumption, but these are third-party services.

6: Based on your evaluation, your decision is:

✓ **A) RECOMMENDED**
B) NOT RECOMMENDED
C) RECOMMENDED WITH PRIORITY
D) RECOMMENDED WITH RESTRICTIONS

**Category**	**Partial total**
**Excellence of the proposal**	**36.00**
	**31.68**
	**9.0**
	**9.0**
**Final sum**	**85.68**
**Average considered the global rating value**	**8.57**<br>**(*meaning 85.7%*)**
